# Automated identification of cell-type-specific genes in the mouse brain by image computing of expression patterns

**DOI:** 10.1186/1471-2105-15-209

**Published:** 2014-06-20

**Authors:** Rongjian Li, Wenlu Zhang, Shuiwang Ji

**Affiliations:** 1 Department of Computer Science, Old Dominion University, 23529 Norfolk, VA, USA

## Abstract

**Background:**

Differential gene expression patterns in cells of the mammalian brain result in the morphological, connectional, and functional diversity of cells. A wide variety of studies have shown that certain genes are expressed only in specific cell-types. Analysis of cell-type-specific gene expression patterns can provide insights into the relationship between genes, connectivity, brain regions, and cell-types. However, automated methods for identifying cell-type-specific genes are lacking to date.

**Results:**

Here, we describe a set of computational methods for identifying cell-type-specific genes in the mouse brain by automated image computing of *in situ* hybridization (ISH) expression patterns. We applied invariant image feature descriptors to capture local gene expression information from cellular-resolution ISH images. We then built image-level representations by applying vector quantization on the image descriptors. We employed regularized learning methods for classifying genes specifically expressed in different brain cell-types. These methods can also rank image features based on their discriminative power. We used a data set of 2,872 genes from the Allen Brain Atlas in the experiments. Results showed that our methods are predictive of cell-type-specificity of genes. Our classifiers achieved AUC values of approximately 87% when the enrichment level is set to 20. In addition, we showed that the highly-ranked image features captured the relationship between cell-types.

**Conclusions:**

Overall, our results showed that automated image computing methods could potentially be used to identify cell-type-specific genes in the mouse brain.

## Background

Although all cells in the brain are genetically identical, they can develop into different cell-types that are distinct in morphology, connectivity, and function. For example, the mammalian brain contains an enormous number of neuronal and glial cells. The neuronal cells are responsible for information communication and processing, while the glial cells are traditionally considered to provide supportive functions. Cell-type diversity is resulted from the different sets of molecules that cells of each type contain. This is in turn due to the differential expression and regulation of genes in the genome. Thus, analysis of gene expression patterns provides an informative way of studying cellular diversity [1,2]. In these studies, it has been commonly observed that some genes are specifically expressed in certain cell-types. These genes serve as cell-type markers and might define cell-type-specific transcriptional programs [3,4]. A complete catalogue of the cell-type-specific genes would be valuable in elucidating the relationship between gene expression patterns, connectivity, brain regions, and cell-types [5-9].

Currently, both experimental and computational approaches have been used to study cell-type-specific gene expression patterns. Experimental methods involve in separating cells of different types from heterogeneous tissues and measuring gene expression levels in the separated tissues using microarrays. Along this line, multiple techniques have been developed for tissue processing; they, however, suffer from different limitations [3]. As an alternative approach, current computational methods identify cell-type-specific genes by comparing their expression profiles captured by either microarrays [10-12] or *in situ* hybridization (ISH) voxel-level data [13]. These approaches either lack the fine spatial resolution or the high-order expression characteristics that are needed for resolving cell-type-specificity.

**Figure 1:**
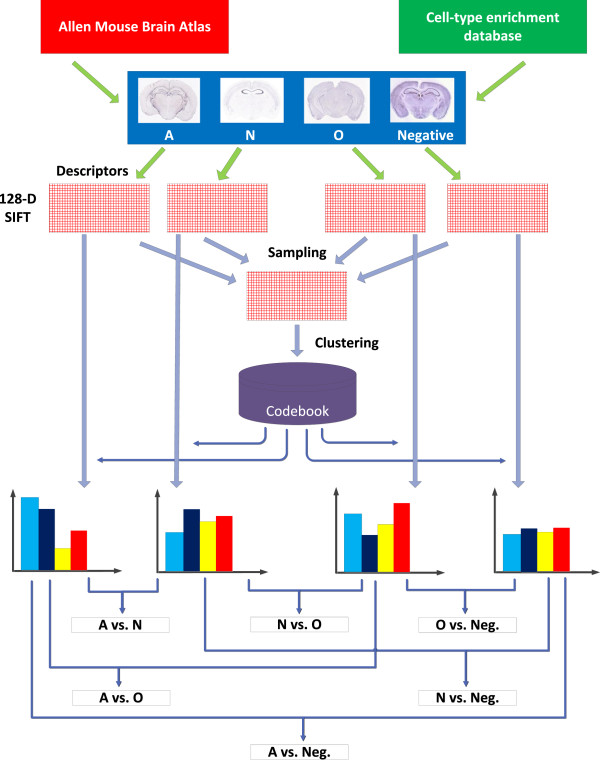
The pipeline of our methods for automated identification of cell-type-specific genes.

In this study, we aimed at identifying cell-type-specific genes by mining and analyzing the high-resolution ISH expression pattern images directly. We applied invariant image feature descriptors to compute high-order expression characteristics from ISH images. These descriptors were computed on dense and overlapping local patches, leading to millions of descriptors from each ISH image section. They collectively capture the local gene expression information, and the spatial information is implicitly encoded into the overlapping patches. To obtain image-level representations, we first clustered these descriptors to obtain the visual words that represent the dominant local expression patterns. We then computed a bag-of-words representation for each ISH image by constructing a histogram based on the visual words. This representation counts the frequency of each visual word occurring in each ISH image, forming a high-level representation of an ISH image. We employed regularized learning methods for discriminating genes specifically expressed in different major brain cell-types, namely, neurons, astrocytes, and oligodendrocytes [1]. Our method can also identify the visual words that are most distinct between different brain cell-types [14]. To obtain a robust estimation of the most discriminative visual words, we employ stability selection to construct an ensemble model. The pipeline of our proposed methods is depicted in Figure 1.

Our results showed that the high-level representations computed directly from cellular-resolution ISH images are predictive of cell-type-specificity of genes in major brain cell types. We used the area under the receiver operating characteristic curve (AUC) as the performance measure [15,16]. We achieved AUC values of approximately 87% in five out of the six tasks when the threshold value for fold enrichment is set to 20, a recommended value based on experimental data [1]. Our results also showed that the image-based invariant representations for ISH images generally yielded better performance than voxel-based features in discriminating genes enriched in different brain cell types. The average AUC value given by our image-based approach on data sets with >1.5 enrichment fold was approximately 75% while an average AUC value of 65% was achieved by voxel-based features. Visualization of highly-ranked features showed that they corresponded to locations containing the most discriminative features among brain cell-types. We also compared the performance of different tasks to investigate the intrinsic relationship between various brain cell-types. Our results showed that the relative performance differences among various brain cell-types are generally consistent with our current knowledge on cell-type functions.

## Material and methods

### Allen mouse brain atlas

The Allen Mouse Brain Atlas provides genome-wide, three-dimensional, high-resolution *in situ* hybridization (ISH) gene expression images for approximately 20,000 genes in the sagittal section for the 56-day old male mice [17]. In addition, coronal sections at a higher resolution are available for a set of about 4,000 genes showing restricted expression patterns. For each experiment, a set of high-resolution, two-dimensional image series are generated. These image slices are subsequently processed by an informatics data processing pipeline to generate grid-level voxel data in the Allen Reference Atlas space [18]. The output of the pipeline is quantified expression values at a grid voxel level [19,20]. The voxel-level data have been used to identify cell-type-specific genes based on correlation search [13]. Note that the selection of coronal genes was biased toward genes enriched in cortical and/or hippocampal regions [21].

### ISH image feature extraction

To fully exploit the cellular-resolution ISH images and extract high-order information for classification, we computed features from the original ISH images directly. The ISH images we used were taken from different mouse brains. Thus, the shape and size of the brain and various anatomical structures might vary from image to image. Additionally, tissue processing and image acquisition might also introduce distortions on the images. To account for these image-level variations, we employed the scale-invariant feature transform (SIFT) descriptor to capture expression patterns on local patches of ISH images [22,23]. This approach can produce robust representations that are invariant to various distortions on the images. To compute SIFT features, an image is first convolved with a sequence of Gaussian filters of different scales to produce difference-of-Gaussian (DOG) images. Stable key-point locations are then detected from these DOG images. A set of orientation histograms on 4×4 neighborhoods at each location are subsequently computed, and each histogram contains 8 spatial bins recording the pixel gradients in 8 orientations.

In many of the current image classification systems, key-point extractors are typically not used [24,25]. Instead, SIFT features are commonly applied on regularly spaced grid on the images, leading to densely populated SIFT descriptors. Following [26,27] we also applied dense SIFT features on the ISH images [28]. This generated approximately 1 million SIFT feature vectors from each ISH image section [26]. In our work, we used the most medial slice of each sagittal section image series. For the coronal section image series, we used the slice with the median Section ID that corresponds to the middle location between the most posterior section showing the cerebellum and hindbrain and the most anterior section showing the olfactory bulb. The use of more slices would incur high computational cost. In addition, it has been shown in [26] that performance may not be improved when more slices were used. In the Allen Mouse Brain Atlas, a detection algorithm was applied to each ISH image to create a mask identifying pixels in the ISH image that correspond with gene expression. Thus foreground pixels are considered to correspond with gene expression while background pixels are not [17]. Only the SIFT descriptors computed from the foreground pixels were used in our study.

### High-level feature construction

In order to derive an image-level representation for cell-type-specific gene classification, we employed the bag-of-words method to construct ISH image representations [29-31]. To construct a visual codebook, we randomly sampled the non-zero descriptors of every image to obtain a descriptor pool of size 100,000. In some of the classification tasks, the numbers of images in the two classes differ significantly. To take this situation into account, we equalized the number of descriptors chosen from both classes. That is, approximately half of the sampled descriptors were from each of the two classes. The descriptors from each class were equally distributed among all images in that class.

We applied the *K*-means algorithm to cluster the SIFT descriptors in this pool. Since the *K*-means algorithm depends on the initialization, we repeated the algorithm three times with random initializations and used the one with the smallest summed within-cluster distance. The cluster centers were considered as “visual words” in the codebook. We then represented an entire image as a global histogram counting the number of occurrences of each visual word in the codebook. The size of the resulting histogram is equal to the number of words in the codebook, which is also the number of clusters used in the clustering algorithm.

Formally, let ${\text{c}}_{1},\ldots ,{\text{c}}_{m}\in R^{d}$ be the *m* cluster centers (visual words), and let ${\text{v}}_{1},\ldots ,{\text{v}}_{n}\in R^{d}$ be the *n* SIFT features extracted from an image, where *d* = 128 for SIFT. Then the bag-of-words representation **x** is *m*-dimensional, and the *k*-th component *x*_*k*_ of **x** is computed as

$$x_{k}=\overset{n}{\underset{i=1}{\sum }}\delta \left(k,\arg\underset{j}{\min}||{\text{v}}_{i}-{\text{c}}_{j}||\right),$$

where *δ*(*a*,*b*)=1 if *a*=*b*, and 0 otherwise, and ||·|| denotes the vector *ℓ*_2_-norm.

To capture the spatial expression patterns at different scales, we constructed four separate codebooks for images with four different resolutions. We then quantized each image using multiple bags of visual words, one for each resolution. The representations for different resolutions were then concatenated to form a single representation for the image. Following [26], we fixed the number of clusters to be 500 in the reported results. To account for the zero descriptors, we introduced an extra dimension in the histogram to record the number of zero descriptors for each image at each resolution. Eventually, an ISH image was represented by a high-level feature vector $\text{x}\in R^{p}$, where *p*=(500+1)×4=2004. Note that the bag-of-words representation has been successfully applied to represent biological images in the past [26,32]. In addition, the local binary pattern (LBP) features have been used in [33] to identify genes expressed in cerebellar layers. We have compared the LBP features with the bag-of-words features and observed that the later performed better for the problem studied in this work.

### Cell-type-specific gene classification

**Figure 2:**
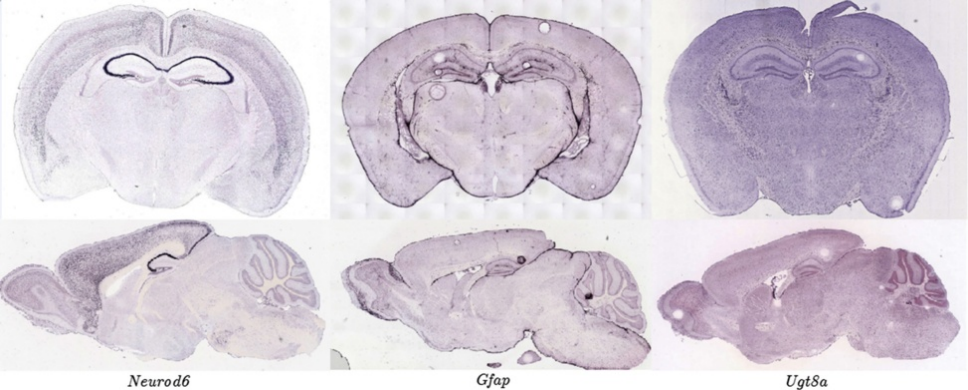
**Sample ISH images for genes *Neurod6*, *Gfap*, and *Ugt8a * that are enriched in neurons, astrocytes, and oligodendrocytes, respectively.** Selected images from approximately the same location were shown for coronal (top) and sagittal (bottom) sections.

We identify the cell-type specificity of genes by classifying the high-level image feature representations constructed above. To achieve this, we need a data set of genes with the corresponding cell-type specificity for training and evaluating our methods. In [1], the fluorescent-activated cell sorting technique was used to isolate and purify the astrocytes, neurons, and oligodendrocytes from the developing mouse forebrain. The expression levels of over 20,000 genes in these cell types were then measured using microarrays, providing a quantitative, genome-wide characterization of the gene expression levels in different brain cell types. By comparing the expression levels of genes across these major brain cell types, three lists of genes enriched in astrocytes, neurons, and oligodendrocytes, respectively, were generated and ranked based on the folds of enrichment. The expression patterns of some example genes enriched in each of the three cell-types are displayed in Figure 2. Note that the data in [1] were obtained from the mouse forebrain, instead of the whole brain.

In this work, we trained and evaluated our methods based on the genes enriched in astrocytes, neurons, and oligodendrocytes [1]. For each gene studied in [1], we checked the availability of ISH images from the Allen Mouse Brain Atlas. By doing this, we obtained a database consisting of 6,660 ISH image series representing 2,872 genes in total. Note that each gene in this database could be associated with more than one cell type, though this does not happen very often.

Each gene in this database is associated with one class label, which is either one of the three cell-types or a negative class label when it does not belong to any of the three classes. To discriminate genes with different class labels, we designed six classification tasks by constructing different positive and negative data samples. In the first three tasks, we used genes enriched in one specific cell-type as positive examples and the negative samples consist of genes with negative class labels. For the other three tasks, we designed classification tasks to discriminate genes enriched in different brain cell-types. Results showed that classification of genes enriched in different brain cell-types yielded insights on the cell-type relationships. The statistics on the numbers of images and genes for these six tasks when the threshold for fold enrichment is 1.5 are given in Table 1. The pipeline of our proposed methods is depicted in Figure 1.

**Table 1:** Statistics on the numbers of images and genes for each of the six tasks with different thresholds for fold enrichment

**Folds**	**Tasks**	**Number of genes**	**Number of images**
1.5	A vs. Neg.	711 vs. 939	775 vs. 981
	N vs. Neg.	775 vs. 939	844 vs. 981
	O vs. Neg.	541 vs. 939	577 vs. 981
	O vs. A	501 vs. 671	532 vs. 730
	A vs. N	690 vs. 754	754 vs. 823
	N vs. O	753 vs. 519	819 vs. 552
10	A vs. Neg.	72 vs. 939	80 vs. 981
	N vs. Neg.	178 vs. 939	209 vs. 981
	O vs. Neg.	47 vs. 939	50 vs. 981
	O vs. A	47 vs. 72	50 vs. 80
	A vs. N	72 vs. 178	80 vs. 209
	N vs. O	178 vs. 47	209 vs. 50
20	A vs. Neg.	26 vs. 939	31 vs. 981
	N vs. Neg.	67 vs. 939	78 vs. 981
	O vs. Neg.	17 vs. 939	18 vs. 981
	O vs. A	17 vs. 26	18 vs. 31
	A vs. N	26 vs. 67	31 vs. 78
	N vs. O	67 vs. 17	78 vs. 18

### Classification and image feature selection

Given a set of training samples ${\left\{{\text{x}}_{i},y_{i}\right\}}_{i=1}^{n}$, where ${\text{x}}_{i}\in R^{p}$ denotes the input feature vector, and *y*_*i*_∈{−1,1} denotes the corresponding output label. In the problem considered in this work, **x**_*i*_ represents the bag-of-words feature vector, and *y*_*i*_ encodes the cell-type enrichment information of the corresponding gene. We employed the following regularized formulation for classification:

(1) $$\underset{\text{w}}{\min}\overset{n}{\underset{i=1}{\sum }}L\left({\text{w}}^{T}{\text{x}}_{i}+b,y_{i}\right)+\lambda \Omega (\text{w}),$$

where $\text{w}\in R^{p}$ and b∈R denote the model weight vector and bias term, respectively, *Ω*(**w**) denotes the regularization term, and *λ* is the regularization parameter.

In this study, we employed the logistic regression loss function as this loss yielded competitive performance in classification tasks [34,35]. The *ℓ*_2_-norm regularization *Ω*(**w**)=∥**w**∥_2_ was used when making predictions [36]. Additionally, we were interested in identifying the most important image features that contributed to the classification performance. This can be achieved by employing the *ℓ*_1_-norm regularization *Ω*(**w**)=∥**w**∥_1_, which drives some entries of **w** to zero, leading to feature selection [37-42].

To make the *ℓ*_1_-norm based feature selection robust and stable, we employed an ensemble learning technique known as stability selection [43,44]. In this technique, a set of *λ* values were selected, and data sets of size ⌊*n*/2⌋ were repeatedly sampled, without replacement, from the original data of size *n*. For each sampled data set, a set of models, corresponding to different *λ* values, were trained. Then the selection probability for each feature under a particular *λ* value was computed as the relative frequency that this feature was selected among the multiple random samples. Finally, the maximum selection probability across the *λ* values was computed and used to rank the features.

## Results and discussion

We formulated the prediction of cell-type-specific genes as a set of six binary-class classification tasks. The prediction was performed by using *ℓ*_2_-norm regularized logistic regression [45]. We also employed the *ℓ*_1_-norm regularized logistic regression [39] and stability selection for image feature ranking. For each prediction task, we used the area under the ROC curve (AUC) as the performance measure [15,16]. We randomly partitioned the entire data set for each task into training and test set so that 2/3 of the data were in the training set, and the remaining 1/3 were in the test set. To obtain robust performance estimation, this random partition was performed 30 times, and the statistics computed over these 30 trials were reported.

In [1], genes with >1.5-fold enrichment were reported for each of the astrocyte, neuron, and oligodendrocyte cell types. It was also stated in [1] that genes enriched with >20-fold should be considered as cell-type-specific based on the enrichment levels of well-established cell type markers. In [4] genes with >10-fold enrichment were considered as cell-type-specific genes. We thus generated multiple data sets by using 1.5, 10, and 20 as cutoff enrichment levels for each of the six tasks. The numbers of genes and images in each task were summarized in Table 1.

In the Allen Mouse Brain Atlas, ISH images are provided in both the sagittal and the coronal sections, and we used only those genes with both coronal and sagittal data. We extracted SIFT features and constructed high-level representations for the coronal and the sagittal images separately. Since images from different sections might capture different and complementary information, we also concatenated the coronal and sagittal representations in the classification tasks. To ensure that all features have the same dimensionality, the codebook size was reduced to 250 so that the concatenated features have the same dimensionality as the features constructed from only coronal and sagittal images. We also used the same set of genes for the coronal and the sagittal images so that the results are directly comparable.

### Performance of cell-type-specific gene identification

**Figure 3:**
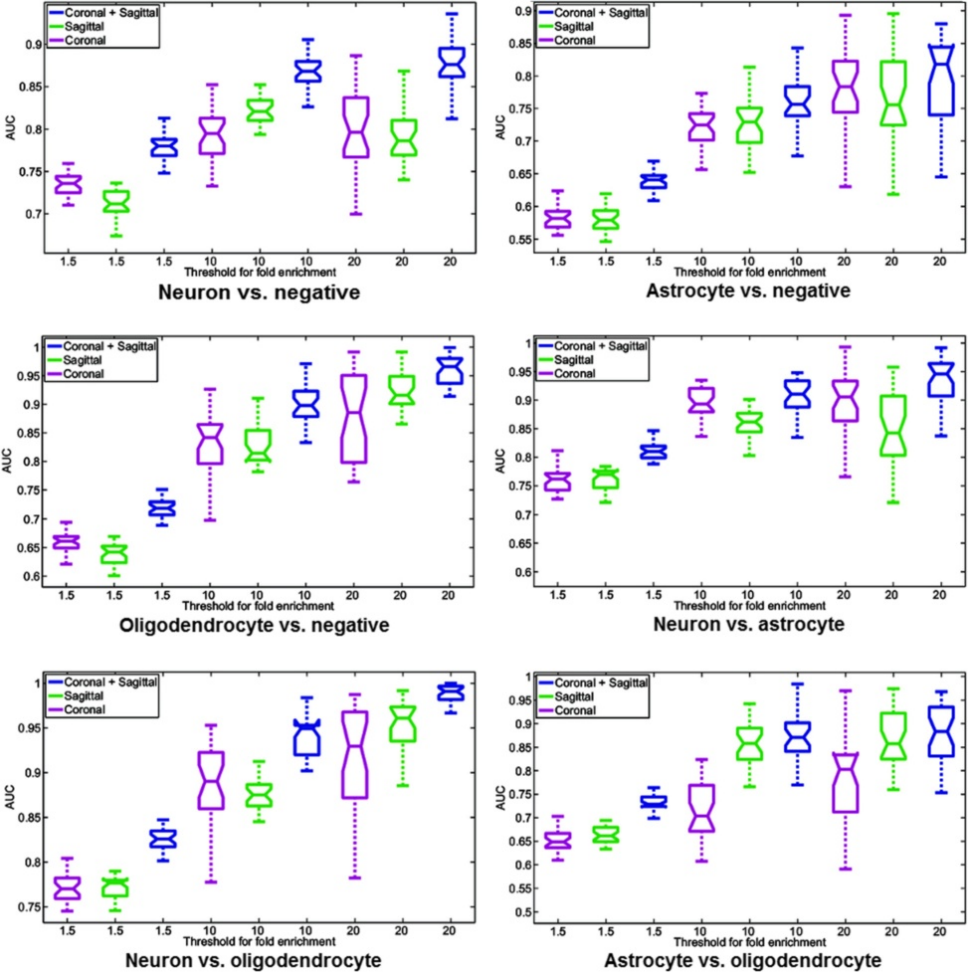
**Box plots of the classification performance achieved on the six tasks.** Each plot corresponds to one of the six tasks, and nine different data sets are generated by using different thresholds for the fold enrichment and different image sections (coronal, sagittal, and coronal + sagittal). For each task, the entire data set is randomly partitioned so that 2/3 of the data is in the training set and the rest 1/3 is in the test set. A total of 30 random partitions are generated. The central mark represents the median, the edges of the box denote the 25th and 75th percentiles. The whiskers extend to the minimum and maximum values not considered outliers, and outliers are plotted individually. The numbers of genes used for different tasks are given in Table 1.

We reported the predictive performance achieved by the proposed methods on different data sets in Figure 3 using box plots. It can be observed from the results that the predictive performance was generally higher on data sets with larger enrichment fold cutoff values. This result is consistent with the fact that genes with large enrichment folds tend to have more cell-type-specificity and thus were easier to identify by our computational methods. In addition, we can observe that combination of the coronal and the sagittal images invariably yielded higher performance than either the coronal or the sagittal images individually, suggesting that different sectional images capture complementary information.

We now consider the performance achieved by the combination of the coronal and sagittal images, as these data sets yielded the best performance. When the enrichment fold cutoff value was set to 1.5, the performance on five out of the six tasks was higher than 0.7. When the cutoff value was increased to 10, the performance on five out of the six tasks reached 0.85. When the cutoff value was further increased to 20, the performance on five out of the six tasks became higher than 0.87. Note that a comparative study in [1] showed that genes enriched with >20-fold should be considered as cell-type-specific. At this level, our proposed methods can achieve high predictive performance. These results demonstrated that our image-based predictive methods were able to identify cell-type-specific genes in major brain cell types.

### Comparison with voxel-based results

The initial attempt to identify cell-type-specific genes from the ISH data used the grid-level voxel data generated from the registered ISH images [13]. In particular, [13] used well-established cell-type marker genes as queries to identify genes enriched in the same cell-type. This was achieved by computing the correlations of all other genes with these marker genes based on the voxel-level expression grid data. A high correlation value was considered as a high probability of enriching in the same cell-type. We compared the voxel-based features and our image-based features in identifying cell-type-specific genes in a discriminative learning framework.

**Figure 4:**
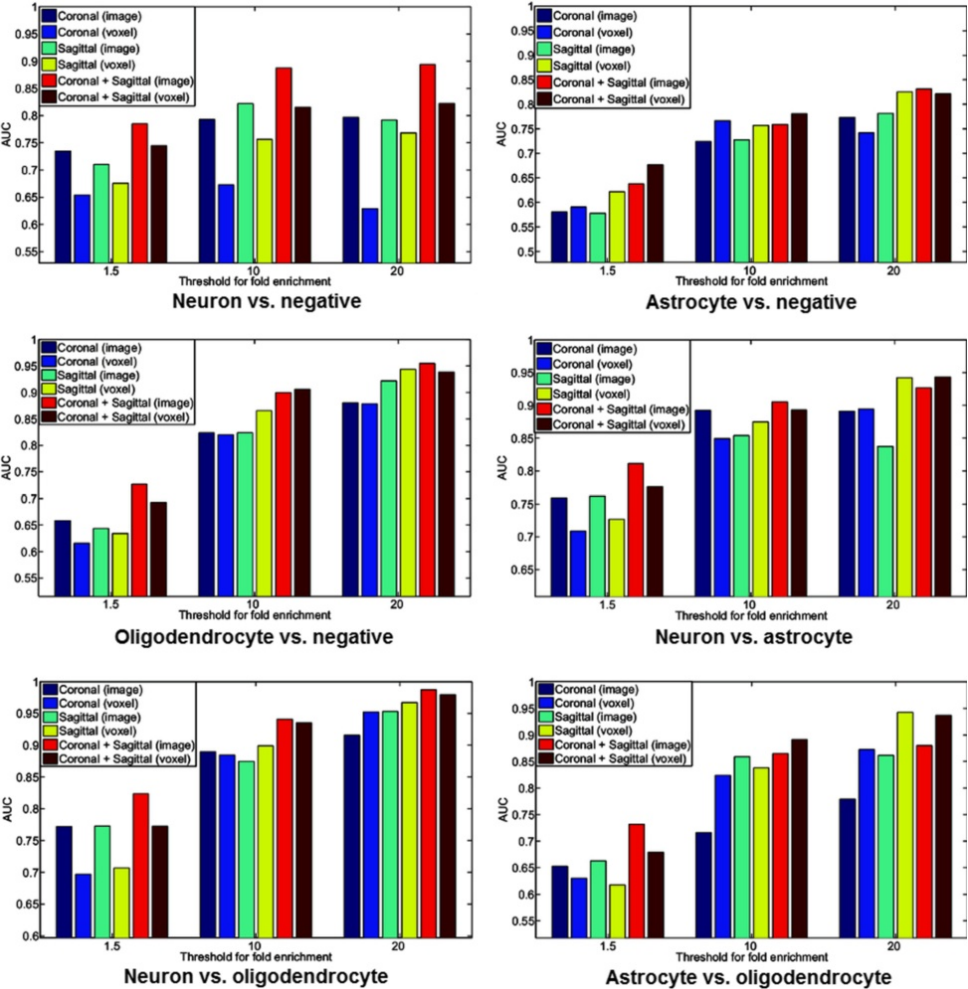
**Comparison of classification performance achieved by our image features and that by the voxel features used in prior work.** The performance on the six tasks are compared, and nine data sets are used for each task. For each task on a specific data set, the entire data set is randomly partitioned so that 2/3 of the data is in the training set and the rest 1/3 is in the test set. A total of 30 random partitions are generated, and the average performance is reported. The numbers of genes used for different tasks are given in Table 1.

**Figure 5:**
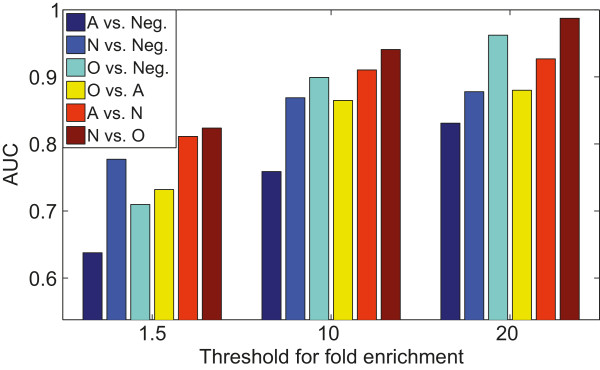
**Comparison of performance achieved on the six different tasks.** We only report the results using combination of coronal and sagittal data, since this data yielded the best performance. The numbers of genes used for different tasks are given in Table 1.

Specifically, we compared the performance of methods using two different types of data, namely the voxel-level expression energy values and the invariant feature representations computed directly from the ISH images. To this end, we used the grid-level expression energy values as features and built discriminative classifiers as we did with our image-based features. That is, we employed the same set of protocols but replaced our image-based features with the voxel-based features where all annotated voxels were used. The results for all six tasks were given in Figure 4. To evaluate the statistical significance of the performance differences, we performed two-sided Wilcoxon signed rank tests on the AUC values produced by 30 random trials, and the *p*-values were reported in Table 2.We can observe from these results that, in the neuron vs. negative classification task, our image-based method significantly outperformed the voxel-based method on all nine data sets. In contrast, these two methods yielded similar performance in classifying astrocyte vs. negative images, and most of the performance differences on this task are statistically not significant. Note that from the results reported below in Figure 5 we can see that the astrocyte vs. negative task gave the lowest overall performance among all six tasks. Thus, it seems that astrocyte-enriched genes are intrinsically difficult to identify, regardless of the feature representations used. For the other four tasks, we observed that our image-based method outperformed voxel-based method consistently and significantly on data sets with >1.5 enrichment fold. For instance, the average AUC value given by our image-based approach was approximately 0.75 while the average AUC value achieved by voxel-based features was approximately 0.65. The performance on other data sets were generally similar, and the differences were mostly not significant. These results demonstrated that our image-based invariant representations were generally better than voxel-based features in discriminating genes enriched in different brain cell types. In addition, the differences were particularly apparent for genes with low cell-type-specificity.

**Table 2:** Statistical test results in comparing our image-based method with voxel-based method

**Folds**	**Sections**	**A vs. Neg.**	**N vs. Neg.**	**O vs. Neg.**	**O vs. A**	**A vs. N**	**N vs. O**
1.5	Coronal	0.0822	**1.7E-06**	**4.7E-06**	**0.0036**	**1.7E-06**	**1.7E-06**
	Sagittal	1.7E-06	**8.5E-06**	0.1306	**2.9E-6**	**1.7E-06**	**2.1E-06**
	Cor.+Sag.	3.5E-06	**1.2E-05**	**0.0017**	**1.7E-06**	**2.6E-06**	**1.7E-06**
10	Coronal	6.6E-04	**1.7E-06**	0.9263	9.3E-06	**8.7E-05**	0.7343
	Sagittal	0.0558	**1.7E-06**	5.5E-4	0.0916	0.0180	0.0052
	Cor.+Sag.	0.0387	**1.1E-05**	0.5038	0.1086	0.3389	0.4908
20	Coronal	**0.0612**	**1.9E-06**	0.9590	0.0001	0.7188	0.0100
	Sagittal	0.0387	**0.0026**	0.0157	2.7E-5	5.7E-6	0.0614
	Cor.+Sag.	0.6435	**9.7E-05**	**0.0114**	4.0E-4	0.3359	**0.0349**

We employed two-sided Wilcoxon signed rank tests on the AUC values produced by 30 random trials, and the *p*-values were reported. We also performed the one-sided statistical test to compare the mean of image-based multiple trials with that of voxel-based method. The bold values indicate tasks on which image-based method outperforms voxel-based method significantly.

### Ranking of image features

**Figure 6:**
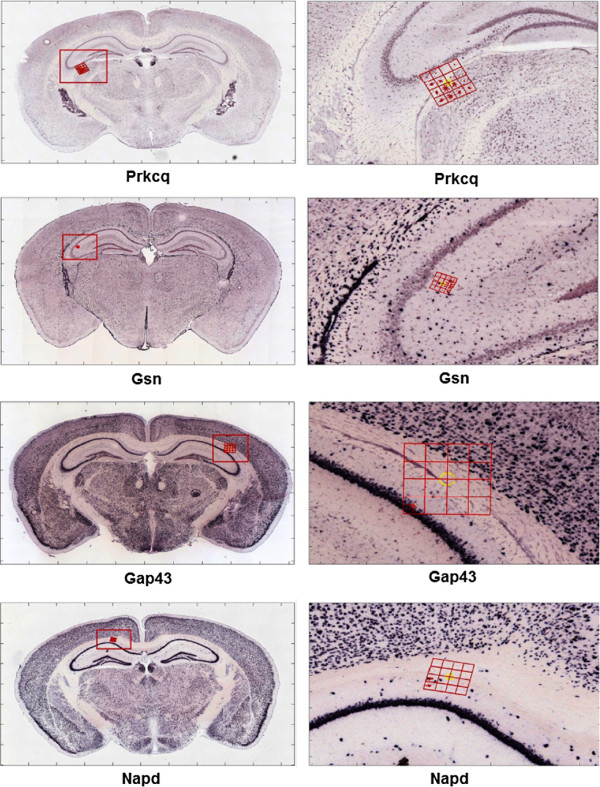
**Visualization of the highly-ranked local image features in discriminating genes enriched in neurons and oligodendrocytes.** For each highly-ranked feature (*i.e.*, cluster center) generated by stability selection, we found the closest SIFT descriptor in the pool and then displayed the corresponding ISH image and the locations on which the SIFT descriptor was computed. The images in the left column are the ISH images along with the SIFT descriptors. The right column shows parts of the ISH images in red boxes on the corresponding image to the left. The grid is used to illustrate the 4 by 4 neighborhoods for the SIFT descriptor. The arrow denotes the direction and the length denotes the magnitude of the orientated histogram.

An appealing property of our regularized learning method is that it can identify the SIFT features and the corresponding image patches that are highly predictive of cell-type enrichment. These highly-ranked features are expected to be located in regions where the most discriminative properties of cell-type enrichment are found, thereby distinguishing the cell-type-specificity of genes accurately. In-depth analysis of these highly-discriminative features might help elucidating the relationships among different brain cell-types. To this end, we obtained and visualized the highly-ranked features for classifying genes enriched in neurons and oligodendrocytes.Specifically, we used stability selection to rank the bag-of-words features, which correspond to the cluster centers of the descriptor pool. Since the cluster centers might not coincide with SIFT features, we located the SIFT features in the pool that were closest to these cluster centers. Finally, we traced back to obtain the ISH images from which these descriptors were extracted. We also recorded the specific locations that these SIFT features were computed and the names of genes corresponding to these ISH images. Some sample highly-ranked features were visualized in Figure 6. We can observe that most of the highly-ranked features identified by our method were indeed located around the boundaries between regions such as hippocampus and isocortex. Additionally, most of these features spanned the boundary between the white matter and the gray matter. It has been widely known that the main function of oligodendrocytes is to provide support and to insulate the axons of neurons. Thus, oligodendrocytes mostly occupy the white matter. In contrast, neurons are mainly located in the gray matter to control information flow within the brain. Therefore, the most discriminative features that distinguish genes enriched in neurons and oligodendrocytes should span the boundary between the gray matter and the white matter. These results demonstrated that our feature ranking method can identify locations in the brain that can distinguish genes enriched in different cell-types, thereby providing insights on the relationships among brain cell-types.

### Performance comparison among different tasks

We observed that the six tasks achieved different performance, and these differences might be related to the intrinsic relationship between various brain cell-types. In order to expedite cross-task comparison, we showed the performance of the six tasks on the combination of coronal and sagittal images in Figure 5. We can see that the relative performance differences among the six tasks are generally consistent across the three data sets with different levels of enrichment.

We can see that the classification of genes enriched in astrocytes versus the negative set yielded the lowest performance on all three data sets. Indeed, astrocytes are among the least-understood brain cells currently, though they account for a high proportion of the brain cells [46]. This type of cells fill the space between neurons and were traditionally considered as providing supportive functions to neurons. However, recent studies showed that thy might control the concentration of extracellular molecules, thereby providing important regulatory functions [46-48]. Thus, the difficulty of distinguishing astrocytes with other cells might be due to the fact that they are spatially very close to other major brain cell-types, and they are found in all areas of the brain [46,48,49].

On the other hand, the classification of genes enriched in neurons and oligodendrocytes yielded the highest performance on all three data sets. Indeed, oligodendrocytes are examples of well-understood glia in the brain. Their primary function was to insulate the axon and thus expedite the transduction of impulses between neurons by creating the myelin sheath [46,48,49]. Thus, oligodendrocytes mainly reside in the white matter, while neurons mainly reside in the gray matter. The spatial complementarity between oligodendrocytes and neurons might explain the relatively high performance of distinguishing genes enriched in these two cell-types.

## Conclusion and outlook

In this study, we aimed at identifying cell-type-specific genes in the mouse brain automatically. This was achieved by combining the high-resolution ISH images from the Allen Brain Atlas with the experimentally-generated lists of genes enriched in astrocytes, neurons, and oligodendrocytes. We constructed invariant, high-level representations from the ISH images directly and employed advanced machine learning techniques to perform the classification and image feature selection. Results showed that our image-based representations were predictive of cell-type enrichment. We also showed that the highly-ranked image features identified by our method explained the intrinsic relationships among brain cell-types. Overall, our results demonstrated that automated image computing could lead to more quantitative and accurate computational modeling and results [50-52].

In the current study, the features for identifying cell-type-specific genes are generic representations and are not trained and tuned to specific tasks. We will explore deep models that are trained end-to-end for fully automated cell-type-specific gene prediction [53,54]. We formulated the cell-type-specific gene identification problem into six separate classification tasks in the current work. However, the prediction of specificity in multiple cell-types might be related. We will employ multi-task learning techniques [55-57] to identify cell-type-specific genes in multiple cell-types simultaneously in the future.

## Competing interests

The authors declare no competing interests.

## Authors’ contributions

SJ conceived the project, RL, WZ, and SJ designed the methodology, RL and WZ performed the experiments, RL and SJ interpreted the results and drafted the manuscript. All authors have read and approved the final manuscript.
